# Supplementary material for: Introducing BPaL: Experiences from countries supported under the LIFT-TB project
Source: PLoS One. 2024 Nov 19;19(11):e0310773. doi: 10.1371/journal.pone.0310773 (PMC11575791; doi:10.1371/journal.pone.0310773)
Supplement: S1 File — (DOCX) [file pone.0310773.s001.docx]

**MINISTRY OF HEALTH OF THE REPUBLIC OF UZBEKISTAN**

**ETHICAL COMMITTEE**

**
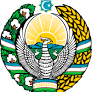
**

**Review Summary**

**Protocol title:** Pilot study to evaluate the effectiveness and safety of the BPaL treatment regimen for rifampicin and fluoroquinolone resistant TB patients and MDR-TB treatment failure/intolerant patients in Uzbekistan

**Dated:** December 2020

**Principal Investigator:** Prof Nargiza Parpieva, Republican specialized scientific and practical medical center of phthisiology and pulmonology, director

**Agenda:** Review of documents presented by the Republican specialized scientific and practical medical center for phthisiology and pulmonology "Pilot study to assess the effectiveness and safety of the treatment regimen for BPAL (using a highly effective combination of drugs Pretomanid-Linezolid-Bedaquiline) in the Republic of Uzbekistan".

**Decision:** Having considered the documents submitted by the Republican specialized scientific and practical medical center for phthisiology and pulmonology, having heard the representative and the reviewer’s conclusion, it is recommended to approve the conduct of an operational study “Pilot study to assess the effectiveness and safety of the treatment regimen for BPAL (using a highly effective combination of drugs Pretomanid-Linezolid-Bedaquiline) in the Republic of Uzbekistan".

**Chairman of the Ethics Committee:** Prof. K.S. Rizaev

**ID:** 1/16-1480

**Date:** 27.02.2021
