# Supplementary material for: Introducing BPaL: Experiences from countries supported under the LIFT-TB project
Source: PLoS One. 2024 Nov 19;19(11):e0310773. doi: 10.1371/journal.pone.0310773 (PMC11575791; doi:10.1371/journal.pone.0310773)
Supplement: S2 File — (DOCX) [file pone.0310773.s002.docx]

**EXTRACT FROM PROTOCOL No. 6**

Meetings of the ethics committee at the research and production association

“Preventive medicine” MoH KR, Bishkek, st. Baytik- Baatyra , 34

Chairman - Bayyzbekova D.A.

Secretary - Mergenova I.O.

July 16, 2020

9 members of the EC took part in the discussion (quorum was reached).

Question No. 1. Ethical examination of packages of documents for scientific research

research work "Evaluation of the effectiveness and safety of the BPaL treatment regimen in

Kyrgyz Republic."

A package of documents for ethical review was received on June 23, 2020.

Applicants: National Center for Phthisiology under the Ministry of Health of the Kyrgyz Republic

Republic. Principal Investigator: Dr Atyrkul Toktogonova, Scientific Department, National Center for Phthisiology.

The primary examination was carried out by a member of the EC - Dooronbekova A.Zh. The following package of documents has been submitted for ethical review:

1) Covering letter of application.

2) Study protocol with relevant annexes (7).

3) Copies of diplomas, certificates, and resumes of members of the research team: 8 people.

Conclusion on the competence of the researcher / team of researchers: Team of researchers are competent researchers because they have previously conducted several public health studies.

Conclusion on the protocol: Protocol for the study “Assessment of the effectiveness and safety of the treatment regimen BPaL in the Kyrgyz Republic". The operational study protocol in question concerns a prospective cohort study using the BPaL treatment regimen. Treatment of extensively drug-resistant TB involves numerous problems for both doctors and national TB control programs due to due to the limited number of available drugs and due to life-threatening the nature of this disease. Experience of using the BPaL regimen for the treatment of patients with XDR-TB is limited and data from a prospective cohort of patients treated with using non-injection longer chemotherapy regimens formulated in according to WHO recommended revised priority classification drugs are not yet available for comparison. However, despite the potential harm, individual patients for whom it is impossible to select an effective chemotherapy regimen according to current recommendations, the BPaL regimen may be beneficial, and it may be used subject to ethical standards. The use of the BPaL regimen for the treatment of such patients will be accompanied by receiving individual informed consent, adequate counseling about potential benefits and harms, as well as active monitoring of development and management of adverse events. Patients should be informed that reproductive toxicity has been documented in animal studies, and that current impact of this chemotherapy regimen on male fertility not sufficiently studied.

The protocol for the study “Assessment of the effectiveness and safety of the treatment regimen

BPaL in the Kyrgyz Republic" complies with good international practices and contains a detailed description of ethical procedures in medical research. Patients who are eligible for inclusion in the study will be provided with information about MDR-TB and the BPaL regimen. Patients will be provided with information on language they understand. Consent for registration will be based on the “Information Sheet patient." Patients will have the opportunity to discuss the Patient Information Sheet with health professional/treatment advocate. Patients will be confident that their decision to participation in the study will not affect the quality of care they receive. As soon as the patient agrees to participate in the pilot project, he will be asked to sign a consent form. All patients who are not eligible to participate in the study refuse to participate or are excluded from the cohort after enrollment will receive MDR-TB treatment at in accordance with national recommendations.

Conclusion on instruments: no comments

Recommendations: None.

Monitoring Agreement: In connection with the COVID-19 epidemiological situation, monitoring visits

are not recommended, therefore the applicant organization must provide a final information and analytical report.

Conclusion: package of documents and research protocol “Evaluation of effectiveness and

safety of the BPaL treatment regimen in the Kyrgyz Republic" corresponds to international ethical standards for conducting research and can be assessed by the ethical committee as "approved".
