# Supplementary material for: Introducing BPaL: Experiences from countries supported under the LIFT-TB project
Source: PLoS One. 2024 Nov 19;19(11):e0310773. doi: 10.1371/journal.pone.0310773 (PMC11575791; doi:10.1371/journal.pone.0310773)
Supplement: S3 File — (ZIP) [file pone.0310773.s003.zip › BPaL ERC approval Uzbekistan.pdf]

O'ZBEKISTON RESPUBLIKASI
SOG'LIQNI SAQLASH VAZIRLIGI
ETIKA QO'MITASI

MINISTRY OF HEALTH
OF REPUBLIC OF UZBEKISTAN
ETHICAL COMMITTE

100015, Toshkent shahar, Oybek ko`chasi, 45- uy Tel.: (998971) 256 -37 -38 256-14-89 Faks: (99871) 256 -45 -04.

№ 1/16-1480

«07» 02 2021 yil

ВЫПИСКА
из протокола №1 заседания Комитета по этике МЗ РУз
от 25 февраля 2021 года (заседание проведено в системе on-line)

Повестка дня:

Рассмотрение документов, представленных Республиканским специализированным научно-практическим медицинским центром фтизиатрии и пульмонологии с просьбой одобрить проведение операционного исследования “Пилотное исследование по оценке эффективности и безопасности схемы лечения BpaL (с применением высокоэффективной комбинации препаратов Претоманид-Линезолид-Бедаквилин) в Республике Узбекистан». База клинического исследования: Республиканский специализированный научно-практический медицинский центр фтизиатрии и пульмонологии.

Решили:

Рассмотрев документы, представленные Республиканский специализированный научно-практический медицинский центр фтизиатрии и пульмонологии заслушав представителя и заключение рецензента, рекомендуется одобрить проведение операционного исследования “Пилотное исследование по оценке эффективности и безопасности схемы лечения BpaL (с применением высокоэффективной комбинации препаратов Претоманид-Линезолид-Бедаквилин) в Республике Узбекистан». База клинического исследования: Республиканский специализированный научно-практический медицинский центр фтизиатрии и пульмонологии.

Председатель Комитета по этике
МЗ РУз, д.м.н. **К.С.Ризаев**

ETIKA QO'MITASI
