# Supplementary material for: Introducing BPaL: Experiences from countries supported under the LIFT-TB project
Source: PLoS One. 2024 Nov 19;19(11):e0310773. doi: 10.1371/journal.pone.0310773 (PMC11575791; doi:10.1371/journal.pone.0310773)
Supplement: S3 File — (ZIP) [file pone.0310773.s003.zip › Indonesia BPaL OR Ethical Approval.pdf]

UNIVERSITAS INDONESIA
FAKULTAS KEDOKTERAN

Gedung Fakultas Kedokteran UI
Jl. Salemba Raya No.6, Jakarta 10430
PO.Box 1358
T. 62.21.3912477, 31930371, 31930373, 3922977, 3927360, 3153236,
F 62 21 3912477, 31930372, 3157288,
E. humas@fk.ui.ac.id, office@fk.ui.ac.id
fk.ui.ac.id

Nomor : KET- 258 /UN2.F1/ETIK/PPM.00.02/2022

KETERANGAN LOLOS KAJI ETIK
ETHICAL APPROVAL

Komite Etik Penelitian Kesehatan Fakultas Kedokteran Universitas Indonesia – RSUPN Dr. Cipto Mangunkusumo dalam upaya melindungi hak asasi dan kesejahteraan subjek penelitian kedokteran, telah mengkaji dengan teliti protokol penelitian yang berjudul:

The Ethics Committee of the Faculty of Medicine, University of Indonesia – Cipto Mangunkusumo Hospital with regards of the Protection of human rights and welfare in medical research, has carefully reviewed the research entitled:

"Penelitian Operasional untuk Mengestimasi Efektivitas dan Keamanan Paduan Pengobatan BPaL (Bedaquiline, Pretomanid, dan Linezolid) untuk Pasien Tuberkulosis Resistan Obat di Indonesia."

Protocol Number : **22-01-0110**

Peneliti Utama / *Principal Investigator* : **Dr. dr. Erlina Burhan, M.Sc., Sp.P(K)**

Nama Institusi / *Name of the Institution* : **Direktorat Pencegahan dan Pengendalian Penyakit Menular Langsung (P2PML) Kementerian Kesehatan RI**

Lokasi Penelitian / *Site* :
1. RSUP Persahabatn
2. RSPI Prof Dr, Sulianti Saroso
3. RSUP Dr. hasan Sadikin
4. RSUD Dr. Soetomo
5. RSUD Dr. Saiful Anwar
6. RSUP Sr Kariadi

Tanggal Persetujuan / *Date of Approval* : 14 MAR 2022
(valid for one year beginning from the date of approval)

Dokumen Disetujui / *Document Approved* :
Proposal Penelitian, Version 0.1 tanggal 17 Desember 2021
Lembar Penjelasan kepada Calon Subjek, Version 0.1 tanggal 24 Desember 2021

dan telah menyetujui protokol berikut dokumen terlampir.
and approves the above mentioned protocol including the attached document.

Ditetapkan di : Jakarta
Specified in

Ketua
Chair

UNIVERSITAS INDONESIA
FAKULTAS KEDOKTERAN

Prof. dr. Rita Sita Sitorus, Ph.D., Sp.M(K)

** Peneliti berkewajiban
1. Menjaga kerahasiaan identitas subjek penelitian.
2. Memberitahukan status penelitian apabila:
   a. Setelah masa berlakunya keterangan lolos kaji etik, penelitian masih belum selesai, dalam hal ini *ethical approval* harus diperpanjang. Harap pengajuan perpanjangan etik dilakukan 2 minggu sebelum masa aktif lolos kaji etik habis.
   b. Penelitian berhenti ditengah jalan.
3. Melaporkan kejadian serius yang tidak diinginkan (*serious adverse events*).
4. Peneliti tidak boleh melakukan tindakan apapun pada subjek sebelum protokol penelitian mendapat lolos kaji etik dan sebelum memperoleh *informed consent* dari subjek penelitian.
5. Menyampaikan laporan akhir, bila penelitian sudah selesai.
6. Cantumkan nomor protokol ID pada setiap komunikasi dengan KEPK FKUI-RSCM.
