# Supplementary material for: Introducing BPaL: Experiences from countries supported under the LIFT-TB project
Source: PLoS One. 2024 Nov 19;19(11):e0310773. doi: 10.1371/journal.pone.0310773 (PMC11575791; doi:10.1371/journal.pone.0310773)
Supplement: S3 File — (ZIP) [file pone.0310773.s003.zip › Kyrgyzstan ERB approval.pdf]

Кыргыз [illegible]
Кыргызская [illegible]
Саламаттыкты сактоо [illegible]
«[illegible] АЛУУЧУ [illegible]ЕДИЦИНА»
Илимий-өндүрүштүк бирикмеси
Министерство здравоохранения
Научно-производственное объединение
«ПРОФИЛАКТИЧЕСКАЯ МЕДИЦИНА»
ИНН 02601199818013
«17» июля 2020 ж.г.
№ 01-3/135
г. Бишкек ш.

ВЫПИСКА ИЗ ПРОТОКОЛА № 6
Заседания этического комитета при научно-производственном объединении «Профилактическая медицина» МЗ КР

г. Бишкек, ул. Байтик- Баатыра, 34   16 июля 2020 г.

Председатель – Байызбекова Д.А.
Секретарь - Мергенова И.О.

В обсуждении приняли участие – 9 членов ЭК (кворум состоялся).
Вопрос № 1. Этической экспертизы пакетов документов на проведение научно-исследовательской работы ««**Оценка эффективности и безопасности схемы лечения BPaL в Кыргызской Республике»»**.
Пакет документов на этическую экспертизу получен 23.06.2020 г.

Заявители: Национальный центр фтизиатрии при Министерстве здравоохранения Кыргызской Республики.

Первичная экспертиза проведена членом ЭК- Дооронбековой А.Ж.
На этическую экспертизу представлен следующий пакет документов:
1) Сопроводительное письмо-заявление.
2) Протокол проведения исследования с соответствующими приложениями (7).
3) Копии дипломов, сертификатов и резюме членов исследовательской команды: 8 человек.

Заключение о компетентности исследователя (команды исследователей) Команда исследователей являются компетентными исследователями, так как ранее ими уже был проведен ряд исследований в области общественного здравоохранения.

Заключение по протоколу.

Протокол проведения исследования **«Оценка эффективности и безопасности схемы лечения BPaL в Кыргызской Республике».**

Рассматриваемый протокол операционного исследования касается проспективного когортного исследования с использованием схемы лечения BPaL.

Лечение ТБ с широкой лекарственной устойчивостью возбудителя сопряжено с многочисленными проблемами как для врачей, так и национальных программ по борьбе с ТБ из-за ограниченного числа доступных лекарственных препаратов и из-за угрожающего жизни характера данного заболевания. Опыт использования режима BPaL для лечения больных ШЛУ-ТБ ограничен, а данные о проспективной когорте больных, получавших лечение с использованием безынъекционных более длительных режимов химиотерапии, составленных в

соответствии с рекомендованной ВОЗ пересмотренной приоритетной классификацией препаратов, пока недоступны для сравнения. Тем не менее, несмотря на потенциальный вред, отдельным пациентам, которым невозможно подобрать эффективный режим химиотерапии в соответствии с современными рекомендациями, режим BPaL может принести пользу, и он может использоваться при условии соблюдения этических стандартов.

Применение режима BPaL для лечения подобных пациентов будет сопровождаться получением индивидуального информированного согласия, адекватным консультированием о потенциальной пользе и вреде, а также активным мониторингом развития и ведением нежелательных явлений. Пациенты должны быть проинформированы о том, что в исследованиях на животных была зафиксирована репродуктивная токсичность, и, что на сегодняшний день влияние данного режима химиотерапии на мужскую фертильность недостаточно изучено.

Протокол проведения исследования «Оценка эффективности и безопасности схемы лечения BPaL в Кыргызской Республике» соответствует надлежащим международным практикам и содержит подробное описание процедур соблюдение этики в медицинских исследованиях.

Пациентам, которые имеют право на включение в исследование, будет предоставлена информация о МЛУ-ТБ и режиме BPaL. Пациентам будет предоставлена информация на понятном им языке. Согласие на регистрацию будет основано на «Информационном листе пациента». Пациенты будут иметь возможность обсудить «Информационный лист пациента» с медицинским работником/сторонником лечения. Пациенты будут уверены, что их решение об участии в исследовании не повлияет на качество получаемой ими помощи. Как только пациент согласится участвовать в пилотном проекте, ему будет предложено подписать форму согласия.

Все пациенты, которые не имеют права на участие в исследовании, отказываются от участия или исключаются из когорты после регистрации, будут проходить курс лечения МЛУ-ТБ в соответствии с национальными рекомендациями.

Заключение по инструментам: замечаний нет

Рекомендации: Нет.

<u>**Соглашение по мониторингу**: В связи с эпидситуацией по COVID-19 мониторинговые выезды не рекомендуются, по этому организация заявитель в обязательном порядке должна предоставить заключительный информационно-аналитический отчет.</u>

Заключение: пакет документов и протокол исследования **«Оценка эффективности и безопасности схемы лечения BPaL в Кыргызской Республике»** соответствуют международным этическим нормам проведения исследований, и могут быть оценены этическим комитетом как *«одобрено»*.

Председатель ЭК,
д.м.н., профессор Д.А.Байызбекова

Секретарь ЭК И.О.Мергенова
