# Supplementary material for: Introducing BPaL: Experiences from countries supported under the LIFT-TB project
Source: PLoS One. 2024 Nov 19;19(11):e0310773. doi: 10.1371/journal.pone.0310773 (PMC11575791; doi:10.1371/journal.pone.0310773)
Supplement: S3 File — (ZIP) [file pone.0310773.s003.zip › SJREB FORM 6 - Notice of Approval The Philippines.pdf]

Republic of the Philippines
Department of Health
SINGLE JOINT RESEARCH ETHICS BOARD

SJREB FORM 6
NOTICE OF APPROVAL

Date: 31 March 2021

This is to certify that the following protocol and related documents have been granted approval by the SJREB for implementation in accordance with the International Conference on the Harmonization of Good Clinical Practice and the National Ethical Guidelines on Health and Health-related Research

SJREB Protocol No.:	SJREB-2021-13	Sponsor Protocol No.:	N/A

Coordinating Investigator:	Dr. Irene A. Flores	Sponsor:	NTP-DOH, TB Alliance, GF-ACCESSTB Project

Title:	Pilot study to estimate the effectiveness and safety of the BPaL treatment regimen in the Philippines

Protocol Version No.:	Protocol OR BPaL PHL version 9.0	Version Date:	March 2021

ICF Version No.:	1. BPaL ICF Tagalog Version 2<br>2. Informed Consent of Parent or guardian English Version 1.0<br>3. Informed Consent of Parent or guardian Tagalog Version 1.0	Version Date:	1. 11 March 2021<br>2. 15 March 2021<br>3. 15 March 2021
Other Documents:			

Members of research team:	1. Dr. Anna Marie Celine Garfin<br>2. Dr. Vincent Balanang
Study sites:	1. Lung Center of the Philippines PMDT TC (LCP)<br>2. Dr. Jose N. Rodriguez Memorial Hospital (DJNRMH)<br>3. Ilocos Training and Regional Medical Center (ITRMC)<br>4. Region I Medical center (R1MC)<br>5. Jose B. Lingad Memorial General Hospital (JBLMGH)<br>6. Batangas Medical Center (BatMC)

	7. Sorsogon Medical MissionGroup Hospital and Health Services (SMMGHHSC)<br>8. Western Visayas Medical Center (WVMC)<br>9. Eversley Childs Sanitarium and General Hospital (ECSGH)<br>10. Zamboanga City Medical Center (ZCMC)<br>11. Xavier University Community Health Care Center (XU-CHCC)<br>12. Southern Philippines Medical Center (SPMC)

Type of Review:	☐ Expedited<br>☑ Full Board<br><br>Meeting date:<br>March 29, 2021	Duration of Approval From – To *(date)*<br><br>March 29, 2021<br>to March 29, 2022	Frequency of continuing review<br><br>Annual

SJREB Chair	Signature	Date
Dr. Jacinto Blas V. Mantaring III	[signature]	April 1, 2021

Investigator Responsibilities after Approval:

- Submit country protocol amendments to the SJREB and site REC for approval before implementing them;
- Submit site-specific amendments to site REC for approval before implementing them;
- Submit annual report for renewal of approval to SJREB;
- Submit SAE and SUSAR reports to the site REC within 7 days;
- Submit progress report every 12 months;
- Submit final report after completion of protocol procedures at the study site;
- Report protocol deviation/violation to the REC study sites;
- Comply with all relevant international and national guidelines and regulations; and
- Abide by the principles of good clinical practice and ethical research

Received by:

Name: ____

Signature: _____________________________________ Date: ____________________________
