# Supplementary material for: Introducing BPaL: Experiences from countries supported under the LIFT-TB project
Source: PLoS One. 2024 Nov 19;19(11):e0310773. doi: 10.1371/journal.pone.0310773 (PMC11575791; doi:10.1371/journal.pone.0310773)
Supplement: S3 File — (ZIP) [file pone.0310773.s003.zip › Translation of ERB approval for BPaL OR in Ukraine.docx]

**CONCLUSION**

**of the Committee on Medical Ethics of the State Institution "National Institute of Phthisiatry and Pulmonology named after F. G. Yanovsky of the National Academy of Medical Sciences of Ukraine" (NIFP NAMSU) on the research materials: "Pilot study to assess the effectiveness and safety of antimycobacterial therapy according to the BPAL regimen in Ukraine"**

The Medical Ethics Committee of the State Institution "F.G. Yanovsky National Institute of Phthisiatry and Pulmonology of the National Academy of Medical Sciences (NAMS) of Ukraine" considered the materials of the study "Pilot study to assess the effectiveness and safety of antimycobacterial therapy according to the BPaL regimen in Ukraine" as part of the research work: "Develop the technology of antimycobacterial therapy using new drugs in patients with drug-resistant pulmonary tuberculosis", which will be conducted in the Drug-resistant Tuberculosis Department of the "F.G. Yanovsky National Institute of Phthisiatry and Pulmonology of the National Academy of Medical Sciences of Ukraine"; Chief researcher - Nataliya Lytvynenko, doctor of Medicine Sciences, head of the drug-resistant tuberculosis department of the government institution "National Institute of Phthisiatry and Pulmonology named after F. G. Yanovsky NAMSU"; co-researchers: Gamazin Yuriy, director of the BPaL/Ukraine project, Organization of optimal technologies in health care; Pogrebna Marina, candidate of Medical Sciences, senior researcher of the department of drug-resistant tuberculosis of the State University "National Institute of Phthisiology and Pulmonology named after F. G. Yanovsky National University"; Senko Yulia, candidate Medical Sciences, senior researcher of the department of drug-resistant tuberculosis of the government institution "National Institute of Phthisiology and Pulmonology named after F. G. Yanovsky NAMSU"; Anastasia Lafeta, researcher of the Drug-resistant Tuberculosis Department of the government institution "National Institute of Phthisiology and Pulmonology named after F. G. Yanovsky NAMSU"; Mirtskhulava Veriko, PhD, Senior Epidemiologist, KNCV Tuberculosis Foundation; Yana Terleeva, Head of the Department of Coordination of Tuberculosis Treatment Programs of the public institution Public Health Center of the Ministry of Health of Ukraine; duration: 07.2020-03.2021 pp.

**The Medical Ethics Committee considered the following:**

1. Application addressed to the chairman of the documents’ reviewing Committee.

2. Research protocol (September 2020, version 4).

3. "Information for the research participant" and "Patient informed consent form for research participation" (appendices 1, 2 of the protocol).

4. Autobiographies of researchers (curriculum vitae).

**The purpose of the study** is to evaluate the effectiveness and safety of treatment according to the BPaL scheme for patients with TB with resistance to rifampicin (Rif-TB) with additional resistance to fluoroquinolones and patients with MDR-TB and documented intolerance/inefficiency of standard treatment regimens (including relapses after the 6 and the 12th month from the end of treatment).

**Main tasks:**

1). To determine efficacy at the time of completion of treatment among patients treated with the BPaL regimen or individualized antimycobacterial therapy regimens.

2). To assess safety based on the incidence of serious adverse reactions (SARs) among patients treated with the BPaL regimen or individualized antimycobacterial therapy regimens.

**Auxiliary tasks**:

1). To determine the time of sputum culture conversion in the case of BPaL treatment regimen or individualized antimycobacterial treatment regimens.

2). To determine the proportion of patients without relapse 6 and 12 months after successful completion of treatment with the ВPaL regimen or individualized antimycobacterial treatment regimens.

3). To determine the proportion of clinically significant adverse events, prolongation of the QT interval, peripheral neuropathy, myelosuppression, optic neuritis, and hepatotoxicity among patients receiving the BPaL regimen or individualized antimycobacterial treatment regimens.

**Aspects of practical implementation** (information regarding the experience of the first use of the BPaL scheme will be published in an international specialized journal and the regime will be included in the national standard medical practice (National Protocol, Standard Operating Procedures [SOP]).

**135 patients** will participate in the study, who will be treated at NIFP NAMSU for 6 months (October 2020 - March 2021), after the study is completed, the decision to expand BPaL treatment in Ukraine will be announced. Patients will be enrolled based on inclusion and exclusion criteria after providing written informed consent.

Research protocol on the introduction of antimycobacterial therapy according to the BPaL regimen in Ukraine, developed by the KNCV Tuberculosis Foundation, National Institute of Phthisiology and Pulmonology named after F.G. Yanovsky NAMSU, the Organization of Optimal Technologies in Health Care (OATH) based on the operational research protocol model published by the Global Initiative to Combat Drug-resistant Tuberculosis (GDI) of the STOP TB Partnership in May 2018 and the ShORRT protocol published by the Special Program on Scientific Research and Training of Specialists in Tropical Diseases (TDR)/World Health Organization (WHO) in April 2020.

This study protocol contains a clear rationale for its conduct and evidentiary information about the proposed drugs. The design of the study, the algorithm for the inclusion of patients, the order of their treatment and follow-up, as well as the detection and management of adverse reactions are described in detail. Data management and monitoring of research implementation, protection of people's rights, which are research object, and indicators are indicated.

Appendix 1 "Information for research participants (booklet)" and Appendix 2 "Form of informed voluntary consent of the patient when performing the protocol" are compiled in accordance with the requirements of the Medical Ethics Commission, i.e. they contain all the necessary information with which research participants should be familiarized (patients).

The researchers' autobiographies (curriculum vitae) show that they all have high professional qualifications.

Therefore, the documents submitted for examination by the Committee on Medical Ethics of the NIFP of NAMSU show that the research is planned to be conducted in compliance with the rights and moral and ethical norms in accordance with the Law of Ukraine "On Medicinal means" and the principles of the Helsinki Declaration.

The Medical Ethics Committee of the State Institution "F.G. Yanovsky National Institute of Phthisiatry and Pulmonology of the National Academy of Sciences of Ukraine" made a positive decision regarding the study: "Pilot study to assess the effectiveness and safety of antimycobacterial therapy according to the regimen of BPaL in Ukraine" within the framework of the research work: "To develop the technology of antimycobacterial therapy using new drugs for patients with drug-resistant tuberculosis of the lungs" in the department of drug-resistant tuberculosis of the "National Institute of Phthisiatry and Pulmonology named after F. G. Yanovsky National Academy of Medical Sciences of Ukraine"; Chief researcher Nataliya Lytvynenko, Doctor of Medicine, Head of the Department of Drug-resistant Tuberculosis of the State Institution "National Institute of Phthisiology and Pulmonology named after F. G. Yanovsky National Academy of Sciences of Ukraine"; co-researchers: Yuriy Gamazin, director of the BPaL/Ukraine project, Organization of optimal technologies in health care; Pogrebna Marina, candidate of Medical Sciences, senior researcher of the Department of drug-resistant tuberculosis of the State Institution "National Institute of Phthisiology and Pulmonology named after F. G. Yanovsky NAMSU"; Senko Yulia, candidate of Medical Sciences, senior researcher of the Department of drug-resistant tuberculosis of the State Institution "National Institute of Phthisiology and Pulmonology named after F. G. Yanovsky NAMSU"; Anastasia Lafeta, researcher of the Drug-resistant Tuberculosis Department of the National Institute of Phthisiology and Pulmonology named after F. G. Yanovsky NAMSU"; Mirtskhulava Veriko, PhD, Senior Epidemiologist, KNCV Tuberculosis Foundation; Yana Terleeva, Head of the Department of Coordination of Tuberculosis Treatment Programs of the Tuberculosis Treatment Programs of the public institution Public Health Center of the Ministry of Health of Ukraine; duration: 07.2020 - 03.2021yy.

The research materials were considered at the meeting of the Committee on Medical Ethics of the State Institution "F.G. Yanovsky National Institute of Phthisiology and Pulmonology of the National Academy of Sciences of Ukraine, which took place on September 24, 2020 (protocol No. 6/2020). The meeting was attended by members of the Committee on Medical Ethics of NIFP NAMSU: Korzhov V.I., Novozhilova I.O., Kalabuha I.A., Rekalova O.M., Puzko T.S., Sergeeva T.A. They voted unanimously to approve the materials of the pilot study submitted for consideration.

Heads of the committee Melnik V.M.
