# Supplementary material for: Introducing BPaL: Experiences from countries supported under the LIFT-TB project
Source: PLoS One. 2024 Nov 19;19(11):e0310773. doi: 10.1371/journal.pone.0310773 (PMC11575791; doi:10.1371/journal.pone.0310773)
Supplement: S3 File — (ZIP) [file pone.0310773.s003.zip › Ukraine ERB approval.PDF]

Комісія з питань етики
ДУ «Національний інститут фтизіатрії і пульмонології ім. Ф.Г. Яновського НАМН України»
ПРИЙНЯТО до розгляду
«21» вересня 2020р.
(підпис Голови / Відповідального секретаря)

ВИСНОВОК
Комітету з медичної етики
Державної установи "Національний інститут фтизіатрії і пульмонології ім. Ф. Г. Яновського НАМН України" (НІФП НАМНУ)
на матеріали дослідження: "Пілотне дослідження для оцінки ефективності та безпеки антимікобактеріальної терапії за режимом BPaL в Україні"

Комітет з медичної етики Державної установи "Національний інститут фтизіатрії і пульмонології ім. Ф. Г. Яновського НАМН України" розглянув матеріали дослідження "Пілотне дослідження для оцінки ефективності та безпеки антимікобактеріальної терапії за режимом BPaL в Україні" в рамках науково-дослідної роботи: «Розробити технологію антимікобактеріальної терапії із використанням нових препаратів у хворих на хіміорезистентний туберкульоз легень», яке буде проводитися у відділі хіміорезистентного туберкульозу ДУ "Національний інститут фтизіатрії і пульмонології ім. Ф. Г. Яновського НАМН України"; Головний дослідник – Литвиненко Наталія, доктор мед. наук, завідувачка відділу хіміорезистентного туберкульозу ДУ «Національний інститут фтизіатрії і пульмонології ім. Ф. Г. Яновського НАМНУ»; співдослідники: Гамазін Юрій, директор проекту BPaL/Україна, Організація оптимальних технологій в охороні здоров'я; Погребна Марина, канд. мед. наук, старший науковий співробітник відділу хіміорезистентного туберкульозу ДУ «Національний інститут фтизіатрії і пульмонології ім. Ф. Г. Яновського НАМНУ»; Сенько Юлія, канд. мед. наук, старший науковий співробітник відділу хіміорезистентного туберкульозу ДУ «Національний інститут фтизіатрії і пульмонології ім. Ф. Г. Яновського НАМНУ»; Лафета Анастасія, дослідниця відділу хіміорезистентного туберкульозу ДУ «Національний інститут фтизіатрії і пульмонології ім. Ф. Г. Яновського НАМНУ»; Міртсхулава Веріко, PhD, старший епідеміолог, Фонд протидії туберкульозу KNCV; Терлеєва Яна, завідувачка відділу координації програм лікування туберкульозу ДУ ЦГЗ МОЗ України; тривалість: 07.2020 – 03.2021 рр.

Комітетом з медичної етики розглянуто наступне:

1. Заява на ім'я голови Комітету про розгляд документів.
2. Протокол дослідження (вересень 2020, версія 4).
3. "Інформація для учасника дослідження" та "Форма інформованої згоди пацієнта на участь у дослідженні" (додатки 1, 2 протоколу).
3. Автобіографії дослідників (curriculum vitae).

Мета дослідження - оцінити ефективність та безпечність лікування за схемою BPaL для хворих на ТБ з резистентністю до ріфампіцину (Риф-ТБ) з додатковою резистентністю до фторхінолонів та пацієнтів з МЛС-ТБ з задокументованою непереносимістю/неефективністю стандартних режимів лікування (включаючи рецидиви після 6-го та 12-го місяця від завершення лікування).

Основні завдання:

1). Визначити ефективність на момент завершення лікування серед пацієнтів, що лікувались за режимом BPaL або індивідуалізованими режимами АМБТ.

2). Оцінити безпеку на основі частоти серйозних побічних реакцій (СПР) серед пацієнтів, що лікувались за режимом BPaL або індивідуалізованими режимами АМБТ.

Допоміжні завдання:

1). Визначити час конверсії культури мокротиння у разі лікування за режимом BPaL або індивідуалізованими режимами АМБТ.

2). Визначити частку пацієнтів без рецидиву через 6 та 12 місяців після успішного завершення лікування за режимом BPaL або індивідуалізованими режимами АМБТ.

3). Визначити частку побічних явищ, що мають клінічне значення, подовження інтервалу QT, периферичну нейропатію, мієлосупресію, неврит зорового нерву та гепатотоксичність серед пацієнтів, які отримували режим BPaL або індивідуалізовані

режими АМБТ.

Аспекти практичного впровадження (інформація стосовно досвіду першого астосування схеми BPaL буде опублікована у міжнародному спеціалізованому журналі та режим буде включений після цього у національну стандартну медичну практику (Національний протокол, стандартні операційні процедури (SOP)).

В дослідженні візьме участь **135 пацієнтів**, які будуть проходити лікування у НІФП НАМНУ протягом 6 місяців (жовтень 2020 р. - березень 2021 р.), після завершення дослідження буде повідомлено про рішення розширення лікування BPaL в Україні. Пацієнти будуть зараховані на основі критеріїв включення та виключення після надання письмової інформованої згоди.

Протокол дослідження з впровадження антимікобактеріальної терапії за режимом BPaL в Україні, розроблений Фондом протидії туберкульозу KNCV, Національним інститутом фтизіатрії та пульмонології імені Ф.Г. Яновського (НАМНУ), Організацію оптимальних технологій в охороні здоров'я (OATH) на основі моделі протоколу операційних досліджень, опублікованої Глобальною ініціативою по боротьбі з хіміорезистентним туберкульозом (GDI) Партнерства "СТОП ТБ" у травні 2018 року[1] та протоколу ShORRT, опублікованого Спеціальною програмою з наукових досліджень і підготовки спеціалістів в області тропічних захворювань (TDR)/Всесвітньою організацією охорони здоров'я (ВООЗ) у квітні 2020 року.

Даний протокол дослідження містить чітке обгрунтування щодо його проведення та доказову інформацію щодо запропонованих препаратів. Детально описано дизайн дослідження, алгоритм включення пацієнтів, порядок їх лікування та подальшого спостереження, а також виявлення та ведення побічних реакцій. Зазначено управління даними та моніторинг виконання дослідження, захист прав людей, що є об'єктами дослідження та індикатори.

Додаток 1 "Інформація для учасника наукового дослідження (буклет)" та додаток 2 "Форма інформованої добровільної згоди пацієнта при виконанні протоколу" складені відповідно до вимог Комісії з медичної етики, тобто містять усю необхідну інформацію з якою з якою мають бути ознайомлені учасники наукового дослідження (пацієнти).

Автобіографії дослідників (curriculum vitae) свідчать, що всі вони мають високу професійну кваліфікацію.

Отже, надані для експертизи Комітету з медичної етики НІФП НАМНУ документи свідчать, що дослідження планується проводити з дотриманням прав та морально-етичних норм відповідно до Закону України "Про лікарські засоби" та принципів Гельсінської декларації.

Комітет з медичної етики Державної установи "Національний інститут фтизіатрії і пульмонології ім. Ф. Г. Яновського НАМН України" ухвалив **позитивне рішення** щодо проведення дослідження: "Пілотне дослідження для оцінки ефективності та безпеки антимікобактеріальної терапії за режимом BPaL в Україні" в рамках науково-дослідної роботи: «Розробити технологію антимікобактеріальної терапії із використанням нових препаратів у хворих на хіміорезистентний туберкульоз легень» у відділі хіміорезистентного туберкульозу ДУ "Національний інститут фтизіатрії і пульмонології ім. Ф. Г. Яновського НАМН України"; Головний дослідник – Литвиненко Наталія, доктор мед. наук, завідувачка відділу хіміорезистентного туберкульозу ДУ «Національний інститут фтизіатрії і пульмонології ім. Ф.Г. Яновського НАМНУ»; співдослідники: Гамазін Юрій, директор проекту BPaL/Україна, Організація оптимальних технологій в охороні здоров'я; Погребна Марина, канд. мед. наук, старший науковий співробітник відділу хіміорезистентного туберкульозу ДУ «Національний інститут фтизіатрії і пульмонології ім. Ф. Г. Яновського НАМНУ»; Сенько Юлія, канд. мед. наук, старший науковий співробітник відділу хіміорезистентного туберкульозу ДУ «Національний інститут фтизіатрії і пульмонології ім. Ф. Г. Яновського НАМНУ»; Лафета Анастасія, дослідниця відділу хіміорезистентного туберкульозу ДУ «Національний інститут фтизіатрії і

пульмонології ім. Ф. Г. Яновського НАМНУ»; Міртсхулава Веріко, PhD, старший епідеміолог, Фонд протидії туберкульозу KNCV; Терлеєва Яна, завідувачка відділу координації програм лікування туберкульозу ДУ ЦГЗ МОЗ України; тривалість: 07.2020 – 03.2021 рр.

Матеріали дослідження розглядалися на засіданні Комітету з медичної етики Державної установи "Національний інститут фтизіатрії і пульмонології ім. Ф. Г. Яновського НАМН України, яке відбулося 24 вересня 2020 р. (протокол № 6/2020). На засіданні були присутні члени Комітету з медичної етики НІФП НАМНУ: Коржов В.І., Новожилова І.О., Калабуха І.А., Рекалова О.М., Пузько Т.С., Сергеєва Т.А.

За затвердження наданих до розгляду матеріалів пілотного дослідження проголосували одноголосно.

Голова Комітету Мельник В. М.

Київ Україна Державна установа "НАЦІОНАЛЬНИЙ ІНСТИТУТ ФТИЗІАТРІЇ І ПУЛЬМОНОЛОГІЇ ІМ. Ф.Г.ЯНОВСЬКОГО НАЦІОНАЛЬНОЇ АКАДЕМІЇ МЕДИЧНИХ НАУК УКРАЇНИ" Ідентифікаційний код 02011964
