# Supplementary material for: Introducing BPaL: Experiences from countries supported under the LIFT-TB project
Source: PLoS One. 2024 Nov 19;19(11):e0310773. doi: 10.1371/journal.pone.0310773 (PMC11575791; doi:10.1371/journal.pone.0310773)
Supplement: S3 File — (ZIP) [file pone.0310773.s003.zip › Uzbekistan ERB approval translation.docx]

**Extract from the minutes No. 1 of the meeting of the Ethics Committee of the Ministry of Health of the Republic of Uzbekistan dated February 25, 2021 (the meeting was held online)**

**Agenda:**

Review of documents presented by the Republican Specialized Scientific and Practical Medical Center for Phthisiatry and Pulmonology "Pilot study to assess the effectiveness and safety of the treatment regimen for BPaL (using a highly effective combination of drugs Pretomanid-Linezolid-Bedaquiline) in the Republic of Uzbekistan." Clinical research base: Republican Specialized Scientific and Practical Medical Center for Phthisiatry and Pulmonology.

Decision:

Having considered the documents submitted by the Republican Specialized Scientific and Practical Medical Center for Phthisiatry and Pulmonology, having heard the representative and the reviewer’s conclusion, it is recommended to approve the conduct of an operational study “Pilot study to assess the effectiveness and safety of the treatment regimen for BPAL (using a highly effective combination of drugs Pretomanid-Linezolid-Bedaquiline) in the Republic Uzbekistan". Clinical research base: Republican Specialized Scientific and Practical Medical Center for Phthisiatry and Pulmonology.
