# Supplementary material for: Introducing BPaL: Experiences from countries supported under the LIFT-TB project
Source: PLoS One. 2024 Nov 19;19(11):e0310773. doi: 10.1371/journal.pone.0310773 (PMC11575791; doi:10.1371/journal.pone.0310773)
Supplement: S3 File — (ZIP) [file pone.0310773.s003.zip › Vietnam BPaL OR MOH ERB approval.pdf]

BỘ Y TẾ	CỘNG HOÀ XÃ HỘI CHỦ NGHĨA VIỆT NAM<br>**Độc lập - Tự do - Hạnh phúc**
Số: 3531/QĐ-BYT	*Hà Nội, ngày 21 tháng 07 năm 2021*

QUYẾT ĐỊNH
Về việc phê duyệt đề cương nghiên cứu thử nghiệm lâm sàng

BỘ TRƯỞNG BỘ Y TẾ

Căn cứ Nghị định số 75/2017/NĐ-CP ngày 20/6/2017của Chính phủ quy định chức năng, nhiệm vụ, quyền hạn và cơ cấu tổ chức của Bộ Y tế;

Căn cứ Thông tư số 29/2018/TT-BYT ngày 29/10/2018 của Bộ Y tế quy định về thử thuốc trên lâm sàng;

Căn cứ Thông tư số 04/2020/TT-BYT ngày 05/3/2020 của Bộ Y tế Quy định việc thành lập, chức năng, nhiệm vụ, quyền hạn của Hội đồng đạo đức trong nghiên cứu y sinh học;

Theo đề nghị của Cục trưởng Cục Khoa học công nghệ và Đào tạo, Bộ Y tế.

QUYẾT ĐỊNH:

Điều 1. Phê duyệt đề cương nghiên cứu thử nghiệm lâm sàng giai đoạn IIIb:

1. Tên nghiên cứu: Nghiên cứu thử nghiệm lâm sàng pha IIIb, không có nhóm chứng đánh giá hiệu quả và tính an toàn của phác đồ điều trị BpaL tại Việt Nam.

2. Giai đoạn nghiên cứu: Giai đoạn IIIb.

3. Nghiên cứu viên chính: PGS.TS Nguyễn Viết Nhung, ThS. BS Phạm Vũ Thường, DS.CKII. Nguyễn Thị Mai Trang.

4. Cơ sở thử thuốc trên lâm sàng: Bệnh viện Phổi Trung ương, Bệnh viện Phổi Hà Nội, Bệnh viện Phạm Ngọc Thạch (TP.Hồ Chí Minh).

5. Nhà tài trợ: Quỹ toàn cầu phòng chống lao giai đoạn 2021-2023 và Hội chống lao Hoàng gia Hà Lan giai đoạn 2020-2025.

6. Địa điểm thu tuyển bệnh nhân: Bệnh viện Phổi Trung ương, Bệnh viện Phổi Hà Nội, Bệnh viện Phạm Ngọc Thạch (TP.Hồ Chí Minh), Bệnh viện Lao và Bệnh Phổi Cần Thơ.

7. Đối tượng nghiên cứu: Bệnh nhân ≥18 tuổi trở lên tại thời điểm tham gia nghiên cứu, được chẩn đoán mắc lao có bằng chứng vi khuẩn xác định bằng nuôi cấy hoặc xét nghiệm phân tử trong vòng 3 tháng tính đến thời điểm sàng lọc hoặc tại thời điểm sàng lọc, đáp ứng tất cả tiêu chuẩn lựa chọn và không có bất kỳ tiêu chuẩn loại trừ nào theo đề cương nghiên cứu.

8. Số lượng đối tượng dự kiến: 567 bệnh nhân.

9. Thời gian thực hiện: 2021-2025.

10. Kinh phí dự toán: 8.843.960.500 (Tám tỷ tám trăm bốn mươi ba triệu chín trăm sáu mươi nghìn năm trăm đồng).

11. Nguồn kinh phí: Quỹ toàn cầu phòng chống lao giai đoạn 2021-2023 và Hội chống lao Hoàng gia Hà Lan giai đoạn 2020-2025.

Điều 2. Thủ trưởng Cơ sở thử thuốc trên lâm sàng có trách nhiệm bảo đảm đáp ứng nguyên tắc, tiêu chuẩn thực hành tốt thử thuốc trên lâm sàng (GCP) trong suốt quá trình thực hiện nghiên cứu. Chủ tịch Hội đồng đạo đức trong nghiên cứu y sinh cấp cơ sở có trách nhiệm theo dõi, giám sát việc tuân thủ đề cương nghiên cứu đã được phê duyệt, đánh giá việc ghi nhận, xử lý, báo cáo, theo dõi biến cố bất lợi xảy ra trong quá trình nghiên cứu. Nghiên cứu viên chính và nhóm nghiên cứu có trách nhiệm thực hiện nghiên cứu theo đúng đề cương nghiên cứu đã được phê duyệt và các quy định hiện hành. Nhà tài trợ có trách nhiệm quản lý, hỗ trợ, giám sát việc thực hiện nghiên cứu bảo đảm chất lượng nghiên cứu và tính xác thực của dữ liệu nghiên cứu.

Điều 3. Thủ trưởng Cơ sở thử thuốc trên lâm sàng, Chủ tịch Hội đồng đạo đức trong nghiên cứu y sinh cấp cơ sở, Nghiên cứu viên chính và Nhà tài trợ chịu trách nhiệm thực hiện đúng mọi quy định của Nhà nước về hoạt động khoa học công nghệ, hợp đồng dịch vụ nghiên cứu, chi tiêu tài chính; tuân thủ theo đúng các hướng dẫn về GCP và các quy định hiện hành liên quan, đảm bảo an toàn tuyệt đối cho các đối tượng tham gia nghiên cứu.

Điều 4. Quyết định này có hiệu lực kể từ ngày ký ban hành.

Điều 5. Các Ông, Bà Cục trưởng Cục Khoa học công nghệ và Đào tạo, Chủ tịch Hội đồng đạo đức trong nghiên cứu y sinh học quốc gia, Thủ trưởng Cơ sở thử thuốc trên lâm sàng và thủ trưởng các đơn vị liên quan chịu trách nhiệm thi hành quyết định này.

Nơi nhận:
- Như điều 5;
- Bộ trưởng (để báo cáo);
- SYT: Hà Nội, TP.HCM (để p/h);
- Nhà tài trợ (để th/hiện);
- Lưu: VT, K2ĐT (02).

KT. BỘ TRƯỞNG
THỨ TRƯỞNG

Trần Văn Thuấn
