# Supplementary material for: Introducing BPaL: Experiences from countries supported under the LIFT-TB project
Source: PLoS One. 2024 Nov 19;19(11):e0310773. doi: 10.1371/journal.pone.0310773 (PMC11575791; doi:10.1371/journal.pone.0310773)
Supplement: S3 File — (ZIP) [file pone.0310773.s003.zip › Vietnam ERB approval translation.docx]

| **MINISTRY OF HEALTH**    No: 3531/QD-BYT | **SOCIALIST REPUBLIC OF VIETNAM**  **Independence - Freedom - Happiness**    *Hanoi, July 21, 2021* |
| --- | --- |

**DECISION**

**On the approval of clinical trial study protocol**



**MINISTER OF HEALTH**

*Pursuant to Decree No. 75/2017/ ND-CP dated June 20, 2017 of the Government regulating the functions, tasks, powers and organizational structure of the Ministry of Health;*

*Pursuant to Circular No. 29/2018/ TT-BYT dated October 29, 2018 of the Ministry of Health regulating clinical drug trials;*

*Pursuant to Circular No. 04/2020/ TT-BYT dated March 5, 2020 of the Ministry of Health regulating the establishment, functions, tasks, and powers of the Ethics Committee;*

*At the request of the Director of the Department of Science, Technology and Training, Ministry of Health.*

**DECIDES:**

**Article 1**. To approve the clinical trial study protocol, phase IIIb:

1. Study name: Phase IIIb, without control group, clinical trial study to evaluate the efficacy and safety of the BPaL treatment regimen in Vietnam.
2. Study phase: Phase IIIb.
3. Principal investigators: Associate Professor. Nguyen Viet Nhung, MD, PhD, Pham Huu Thuong, MD, MPH, Nguyen Thi Mai Trang, Phar, PhD.
4. Clinical trial sites: National Lung Hospital, Hanoi Lung Hospital, Pham Ngoc Thach Hospital (Ho Chi Minh City).
5. Sponsors: Global Fund to Fight Tuberculosis for the period of 2021-2023 and KNCV Tuberculosis Foundation for the 2020-2025 period.
6. Patient recruitment sites: National Lung Hospital, Hanoi Lung Hospital, Pham Ngoc Thach Hospital (Ho Chi Minh City), Can Tho TB and Lung Disease Hospital.
7. Study subjects: Patients from 18 years and older at the time of study participation, diagnosed with TB with bacterial confirmation by culture or molecular testing within 3 months up to the time of screening or at the time of screening, meet all inclusion criteria and do not have any exclusion criteria according to the study protocol.
8. Expected number of subjects: 567 patients.
9. Implementation time: 2021-2025.
10. Estimated budget: 8,843,960,500 (Eight billion eight hundred forty-three million nine hundred sixty thousand five hundred dong).
11. Funding source: Global Fund to Fight Tuberculosis for the period of 2021-2023 and KNCV Tuberculosis Foundation for the 2020-2024 period.

**Article 2.** The head of the clinical trial sites is responsible for ensuring that principles and standards of good clinical practice (GCP) are met throughout the study process. The Chairman of the Ethics Committee of the National Lung Hospital is responsible for monitoring and supervising compliance with the approved study protocol, evaluating the recording, processing, reporting, and monitoring of adverse events that might occur during the study. The principal investigators and research team are responsible for conducting the study in accordance with the approved study protocol and current regulations. The sponsors are responsible for managing, supporting, and supervising study implementation to ensure research quality and authenticity of research data.

**Article 3.** The Head of the clinical sites, Chairman of the Ethics Committee of the National Lung Hospital, principal investigators and sponsors are responsible for properly implementing all state regulations on science and technology activities, research service contracts, financial expenditures; comply with GCP instructions and relevant regulations, ensuring absolute safety for study participants.

**Article 4**. This Decision takes effect from the date of signing.

**Article 5**. Directors of the Department of Science, Technology and Training, Chairman of the National Ethics Committee, Heads of clinical sites and Heads of relevant units are responsible for implementing this decision.

| ***Recipients:***   - As in Article 5; - Minister (to report); - DOH: Hanoi, Ho Chi Minh City (to collaborate); - Sponsors (to implement); - Achieve: Record room, K2DT (02). | **PP. MINISTER**  **DEPUTY MINISTER**  **(Signed and stamped)**  **Tran Van Thuan** |
| --- | --- |
