# Supplementary material for: Introducing BPaL: Experiences from countries supported under the LIFT-TB project
Source: PLoS One. 2024 Nov 19;19(11):e0310773. doi: 10.1371/journal.pone.0310773 (PMC11575791; doi:10.1371/journal.pone.0310773)
Supplement: S3 File — (ZIP) [file Decision Letter BPaL OR Myanmar.pdf › Decision Letter BPaL OR Myanmar.pdf]

Institutional Review Board
Department of Medical Research
No. 5, Ziwaka Road, Dagon Township, Yangon 11191

Letter No: Thakasa (SaTha)2021/002

Date: 30 November 2021

Decision Letter

Project Title: **Pilot study to estimate the effectiveness and safety of the BPaL treatment regimen for multidrug resistant tuberculosis in Myanmar**

Principal Investigator: **Dr Zaw Myint**
Senior TB specialist, NTP central (Yangon branch)
Department of Public Health

Items Accepted:

1. Full Proposal (Amended & Revised) Dated 30th November 2021
2. Informed consent for participants (English & Myanmar version): 11th June 2021
3. Study area: Aung San TB Specialty Hospital and Union TB Institute, Insein Township.
4. Study population: MDR TB patients who are at least 18 years old
5. IRB approval certificate: Ethics/DMR/2021/006 approved on 16 July 2021 (valid up to 15 July 2022)

The Institutional Review Board, Department of Medical Research has accepted the amendments/deviations submitted by the research team and the final decision is to uphold the original ethics approval dated 16 July 2021.

Dr. Khin Thet Wai
Chairperson, Institutional Review Board
Department of Medical Research
