# Supplementary material for: Introducing BPaL: Experiences from countries supported under the LIFT-TB project
Source: PLoS One. 2024 Nov 19;19(11):e0310773. doi: 10.1371/journal.pone.0310773 (PMC11575791; doi:10.1371/journal.pone.0310773)
Supplement: Attachment — Submitted filename: Response to reviewers.docx [file pone.0310773.s004.docx]

**Reviewer #1**

**The authors may consider the below suggestions for their paper:
Background: The WHO recommendation on shorter 9-month treatment regimen (STR) was made in 2016 (**[**https://www.who.int/news/item/12-05-2016-rapid-diagnostic-test-and-shorter-cheaper-treatment-signal-new-hope-for-multidrug-resistant-tuberculosis-patients**](https://www.who.int/news/item/12-05-2016-rapid-diagnostic-test-and-shorter-cheaper-treatment-signal-new-hope-for-multidrug-resistant-tuberculosis-patients)**) but in the background (last sentence) it is mentioned - “Since 2018, the WHO has recommended that for certain MDR/RR-TB.......”
Response:** Thanks to the reviewer for this correction. The text has been amended and now reads as *“Since 2016, the WHO has recommended that for certain MDR-/RR-TB patients, a shorter 9-12 months treatment regimen (STR), with a SLI agent included, could be used instead of a longer (preferably all-oral) regimen.^3^ From 2018 onwards, WHO has considered that a 9-months STR with bedaquiline (Bdq) replacing the SLI agent could be used.^4^”* A new reference 3 has been added and all the subsequent previous references have been renumbered.

**Table 3: Please check the figures and their totals for correctness e.g. there are differences in Philippines between the “number of patients with end of treatment outcome evaluation in REDCap (93)” and “End of treatment success (84)” – is there is any reason why only 84 were evaluated for treatment success while 93 had end of treatment outcome data? Please check the totals (at the bottom row [(82+43+82+34+40) / (86+50+84+39+40) = 279/299 = 93.3%] of the last column with “End of treatment success (%)” which is shown 280/308 (90.9%)
Response:** Differences between the numbers for the number of patients enrolled, end of treatment outcome evaluation in REDCap and end of treatment success, is due to the time factor and exclusions. The data presented is from the ‘REDCap database on 29 January 2024’. This is a dynamic process, with data cleaning, validation and analyses, and numbers have changed since then due to data being added to the database. And although 99 were known to have been enrolled into the BPaL OR patient cohort at that date, this number can change due to “Late Screen failures” as shown in Column 6 of Table 3 and defined in footnote 1. Further changes in the denominator totals in Column 8 “End of treatment success” can occur due to it showing the results of a “ Modified intention to treat (MITT) analysis” with exclusions being made (please refer to footnote 2 for further details). These factors also apply to the totals shown in the bottom row. To stress the dynamic on-going process of the project’s data analyses, the term “interim” has been added to the title “Table 3. Patient enrolment and end of treatment success results (from *interim* analyses of REDCap database), under the BPaL OR component of the LIFT-TB project (REDCap database version of 29 January 2024)”

**Table 4: BPaL safety data showing the numbers with adverse event types but not presenting the numbers requiring decreasing the dose or stop of LZD and the number of patients discontinued treatment. Please specify what types of treatment modifications (dose of Lzd decreased or Lzd stopped or full treatment regimen discontinued?) accounted under “adverse events leading to treatment modification (AELTM)”
Response:** To address this point would almost require a separate manuscript. A multi-country manuscript, including data from all 7 countries, detailing out effectiveness and safety data in detail, is currently being prepared for submission to an international peer-reviewed scientific journal.

**Discussion (the two key changes are): recommended age of patients for the 6-month BPaLM regimen is 14 years and above (it is mentioned in the manuscript ≥15 years) and previous exposure to Bdq, Pa and Lzd defined as 1 month or more (in the manuscript >1-month exposure).**

**Response:** Text amended accordingly

**Reviewer #2**

**Minor comments
- I would suggest revising the term "prove" as OR mayn't be the best option to do so (abstract and 5th para - background).**

**Response:** Text in abstract and 5^th^ paragraph - background has been amended in line with suggestion of Reviewer #2. Now reads in the abstract as *“The OR objectives were to explore the feasibility of introducing the BPaL regimen,… ”* and 5^th^ paragraph – background as “*The primary objectives of the support were to firstly explore the feasibility of introducing the BPaL regimen,…”.*

**- Page 11 1st para, line 3 (Background) - delete "each year" as the year is already specified (2022) in the same sentence.**

**Response:** Text amended.

**- Provide the data on the number of patients who discontinued treatment due to SAEs rather than saying "few" (abstract, and Result page 22). This information (if available) could be added to Table 4 (page 22) as well.**

**Response:** Text amended. Now reads in the abstract as *“… and only 1 patient had to discontinue the complete BPaL treatment regimen*.” and in ‘Results, page 22’ as “*…. only 1 patient had to discontinue the full BPaL regimen…*. As it was just the one patient, the information has been added in the text but not felt necessary to add in Tabe 4 as well.

**- Please clarify why the interim results from Myanmar and Ukraine were not available in Q2/2024 (253 patients? is this related to the contexts in the countries? do you anticipate different outcome?**

**Response:** Myanmar and Ukraine did not use the REDCap database, choosing instead to use an Excel-based database and Epi Info database respectively. The data from these databases were not available as of the time of writing of the manuscript. And when available, required incorporation with the data from the REDCap database, resulting in a much longer time for cleaning, validation and analyses of the data. On current evidence, we do not anticipate any differences in the outcomes from those already reported in this manuscript. A multi-country manuscript, including data from all 7 countries, detailing out effectiveness and safety data in detail, is currently being prepared for submission to an international peer-reviewed scientific journal.

**- In Vietnam, was it due to "very prolonged impact of the COVID-19 pandemic..." or late initiation of the pandemic (unlike in most other countries, the pandemic started in Vietnam in 2021)?**

**Response:** Text amended to clarify the prolonged COVID impact in Vietnam. The main factor was that the responses to the COVID pandemic introduced by the Government in Vietnam (e.g. redeployment of TB staff and TB hospitals to the COVID response were in place for a much longer time than in other countries). Now reads in the Interim Results section, second paragraph as “*The exception was Vietnam (due to a very prolonged impact of the COVID-19 pandemic mainly due to the responses to the COVID pandemic introduced by the Government [e.g. redeployment of TB staff and TB hospitals to the COVID response] were in place for a much longer time than in other countries),…”.*
