# Supplementary material for: Introducing BPaL: Experiences from countries supported under the LIFT-TB project
Source: PLoS One. 2024 Nov 19;19(11):e0310773. doi: 10.1371/journal.pone.0310773 (PMC11575791; doi:10.1371/journal.pone.0310773)
Supplement: Attachment — Submitted filename: Response to reviewers.docx [file pone.0310773.s005.docx]

**6. Review Comments to the Author**

**Please use the space provided to explain your answers to the questions above. You may also include additional comments for the author, including concerns about dual publication, research ethics, or publication ethics. (Please upload your review as an attachment if it exceeds 20,000 characters)**

**Reviewer #1: The authors have addressed my comments raised in a previous round of review which are accepted by me.**

**Reviewer #2: Pease add the information on Vietnam's COVID timeline as the epidemic started late (in 2021) compared to other countries as this might have contributed to the difference in addition to what you have mentioned.**

**Response:** Many thanks to both reviewers for their further comments.

As requested by reviewer #2, information on Vietnam's COVID timeline has been added to the manuscript’s text as footnote 6 on page 12 as follows: “^6^ The COVID-19 pandemic badly impacted the Health Services and the whole of society in Vietnam. All study sites for the screening and enrolment of both DS-TB and DR-TB patients were affected. Regarding screening, there were difficulties in sputum transportation for Xpert and SL LPA (the laboratory staff were overloaded with COVID-19 testing and with COVID-19 illness amongst themselves). Also, patients were reluctant to go to health care facilities due to the fear of COVID-19, the requirement of COVID-19 screening tests, and the difficulties in moving / travelling around during the COVID-19 lockdown period. For enrolment, TB hospitals / facilities and staff were fully or partially redeployed to the COVID-19 response effort (treatment, vaccine, screening), difficulty in requesting the initial inpatient treatment (1 month), and staff themselves falling ill with COVID-19.

Many of the restrictions imposed due to COVID-19 remained in place in Vietnam well into 2022. The first in-person monitoring visits took place only in May 2022. It also took many months of 2022 for the TB services to revert to providing TB services. By then, the issue of enrolment of patients to a competing OR activity had arisen.”
